# Interventional Revascularization of Coronary Artery Lesions in Diabetic Patients; In-hospital and One Year Follow up

**Published:** 2012-12-15

**Authors:** Mohammad Javad Zibaeenezhad, Amir Aslani, Alireza Moniri, Mohammad Ali Kheiri, Seyed Taghi Heydari, Ahmad Amanat, Zahra Daneshvar

**Affiliations:** 1 Cardiovascular Research Center, Shiraz University of Medical Science, Shiraz, IR Iran; 2 Jahrom University of Medical Science, Jahrom, IR Iran

**Keywords:** Diabetes mellitus, Percutaneous Coronary Intervention, Revascularization, Coronary Artery Disease

## Abstract

**Background:**

Diabetes mellitus is a life threatening disease accompanied by several micro- and macro vascular complications. Several modalities are available for interventional revascularization of coronary artery lesions, but their efficacy in diabetic patients is studied only in few patients.

**Materials and Method:**

This study evaluated major in- hospital complications and clinical outcome after one year in 200 consecutive patients who underwent percutaneous Coronary Intervention from 2007 to 2009.

**Results:**

Our findings showed comparable single and 2 vessel stenting, regarding major adverse cardiovascular event in diabetic and nondiabetic patients. In connection with long term and in hospital outcome, no statistically significant difference was found between one and two vessel stenting when drug eluting stent was used in diabetic patients.

**Conclusion:**

The use of drug eluting stent in single or two vessel disease of diabetic patients is technically satisfactory and clinically safe and can substitute for coronary artery bypass grafting.

## 1. Background

Diabetes mellitus (DM) is a chronic and life threatening disease that is induced because of low production of insulin or insensitivity to this substance (1). It is a prevalent disorder and Canadian Community Health Survey (CCHS) had reported DM in 4.9% of Canadian population aged 12 years or more in 2005 (2).

Diabetes has a series of complications of which two main categories are macrovascular problems including stroke, heart disease and microvascular complications such as blindness, renal failure and limb amputation (2). Among macrovascular complications, cardiovascular diseases are associated with high mortality and morbidity (3). Both type I and II of DM are risk factors for coronary artery disease (4) but type I patients presents more cardiovascular diseases in younger subjects than type II (5).

There are several pathophysiologic features of atherosclerosis in diabetic patients. Metabolic and hematologic abnormalities associated with type 2 diabetes include hyperglycemia, insulin resistance, dyslipidemia, inflammation, and thrombophilia (6). Platelets express more Gp IIb/IIIa receptors and are more prone to aggregation, particularly in the presence of hyperglycemia. These abnormalities together contribute to development of hypertension, endothelial cell dysfunction, accelerated atherogenesis and, eventually, coronary thrombosis. Diabetic nephropathy, including reduced creatinine clearance and proteinuria, identifies patients with markedly decreased survival after coronary revascularization (7).

Diabetes not only increases the incidence of coronary disease but also contribute to less favorable prognosis. Although patients with diabetes frequently have concurrent risk factors, diabetes itself is a powerful independent risk factor for cardiovascular events (1).

Macrovascular complications lead to atherosclerosis in coronary, cerebral and peripheral arteries which cause 80% mortality and 75% hospitalization in diabetic patients (3,8). In fact, diabetes alters the function of vascular endothelium, smooth muscle cells and platelets aggravating atherogenesis (9).

Surgical revascularization and percutaneous coronary interventions (PCI) with coronary stents are widely performed for diabetic patients in recent decades, although the outcome of these procedures is unfavorable in diabetic patients (10,11). Several previous studies have shown that angioplasty with drug eluting stents (DES) such as sirolimus eluting stents and paclitaxel eluting stents are more effective compared with bare metal stents and reduce the risk of restenosis and the need for repeating revascularization (9,12). Also in some non- randomized trials, these were shown to reduce the need for repeat revascularization procedures compared with BMS (13,14).

Also DM was defined as past or currently diagnosed disease or requiring medical therapy, according to the American college of Cardiology’s NCDR definition. In patients with chronic total occlusion, DES was superior to bare-metal stents and a preferred treatment in reducing the MACE of patients with diabetes and CTO undergoing PCI (9).

## 2. Materials and methods

This was a non randomized study of patients undergoing PCI in Faghihi hospital affiliated with Shiraz University of Medical Sciences from January 2007 to 2009. MACE, such as unstable angina, myocardial infarction and death was evaluated in- hospital, 6 and 12 months after PCI in all patients. Echocardiography, ETT and dipyridamole technesium scan were used to detect subclinical ischemia in both diabetic and non-diabetic patients (Table1). We used stents diameter sized between 2.25-3.5 mm in both diabetic and non-diabetic groups. The mean of stent length in diabetic and non- diabetic patients was not statistically significant and were 20.96±2.6mm and 20.20 ±2.3mm respectively. Drug-Eluting Stents (DES) used in all diabetic patients were of Paclitaxe kind and the bare metal stents were of different types.

**Table 1:** Diagnostic Measures for Cardiovascular Accidents (Positive MACE).

•	Death because of coronary artery disease during six months and one year after stenting
•	Symptoms of acute infarction defined as presence of one or more of these conditions:
-	New ST-segment elevation
-	New Q-wave
-	3-5 fold increase of serum creatine kinase MB (CKMB) level
-	Positive troponin-I test
-	New onset changes of ECG such as T-wave inversion in addition to a 3-5 fold increase of serum CKMB level
•	Positive exercise test or positive result from cardiac scan with dipyridamole

All the patients had received at least 300-600 mg antiplatlet drug clopidogrel before angioplasty and stenting. After stenting, the patients remained in hospital for 24 hours and underwent emergency angiography if he/ o she had chest pain or new EKG changes such as ST-segment elevation or depression. Patients, without EKG changes were discharged, and given ASA (80-325 mg/day) and clopidogrel (75 mg/day) for at least six months. Anti-ischemic drugs including beta blockers, nitroglycerin, diltiazem and statins were administered as well as antidiabetics depending on clinical status. The patients underwent close follow up and a special nurse was in telephone contact with them and in the meantime had monthly visit with their physicians. However, physicians were accessible to patient by phone, and in clinic if they remained asymptomatic and showed no ECG changes during 6 months and one year follow up evaluation by cardiac scan and B.T.T. Coronary angiography was performed if they became symptomatic or showed signs of ischemia, or had positive E.T.T or showed evidence of ST-T change accompanied with biomarker elevation in clinical fallow up. (Table2) Patients were considered as candidates for repeat PCI or coronary artery bypass grafting (CABG) if a thrombosis and/or restenosis was observed during angiography of stent location or new atherosclerotic lesions were seen in other vessels.

**Table 2:** Indications for Patients' Angiography

symptoms occurring during exercise test
Decrease in blood pressure during exercise test and/or systolic blood pressure below 120 mmHg
ST-segment depression equal or mare than 2 mm and/or ST-segment depression in more than 5 lead and/or ST-segment depression persistent for 5 minutes after stopping the test
ST-segment elevation during exercise test
Chest pain starting in first steps of exercise test (i.e. low exercise work load)
Sustained ventricular tachycardia (VT), symptomatic ventricular arrhythmia and/or non-sustained polymorphic VT with successful resuscitation

Indications of angiography in the first 24 hours:
o ST-T change accompanied by elevated enzyme biomarkers
o ST-T Change accompanied by chest pain suspecting myocardial ischemia

2.1. Statistical analysis

Data included baseline for patient's characteristics, information of coronary angioplasty procedure, and outpatient follow up. Categorical variables are presented as absolute numbers (percent). Continuous data, expressed as mean ± standard deviation, were compared using the student's t-test. The two groups were compared by the chi-square test or Fisher's exact test. Statistical significance was considered as P<0.05.

## 3. Results

The first, patients, including 95 diabetics and 115 non-diabetics, eighty-nine were females and 111 males. Baseline characteristics of the participants are summarized in Table 3. The majority of diabetic patients were females, whereas most non-diabetic subjects were males. Hypertension and hyperlipoproteinemia were significantly more prevalent in the diabetic group, while smoking was less common among them. Single vessel disease prevailed in both groups (80% in diabetics and 71.4% in non-diabetics), followed by two-vessel disease (16.9% in diabetics and 25.7% in non-diabetics). Three-vessel disease was found in approximately 2% of both groups (Table 4).

**Table 3:** Baseline Characteristics of the Study Population

		Diabetic	Non-diabetic
Gender	Male	36	75
	Female	59	30
Mean age		57.12	55.46
Hypertension _b_		56	28
Hyperlipoproteinemia _a_		66	58
Smoker _b_		33	64
Target vessel	LAD	65	76
	RCA	15	23
	LCX	21	18
	Other	3	4

Abbreviations: LAD, left anterior descending artery; RCA, right coronary artery; LCX, left circumflex artery.

^a^P-value < 0.05

^b^P-value < 0.001

**Table 4:** Frequency and Percent of the Numbers of Stented Vessels in Both Groups

Stented Vessel	Diabetic group		Non-diabetic group	
	frequency	Percent	Frequency	Percent
SVS	76	80	75	71.42
2VS	16	16.85	27	25.72
3VS	1	1.05	2	1.91
Others	2	2.1	1	0.95

Table 5 summarizes the incidence of measured outcomes in the two groups. According to this, no statistically important difference was observed between diabetic and non-diabetic groups in positive scan or E.T.T, angiography, acute and sub acute MI, Late MI, CABG, repeat PCI and cardiac death. Event-free survival in all patients was 93.3%. This did not show any statistically significant difference between diabetic and nondiabetic groups (91.6 vs. 94.3%, P=0.35).

**Table 5:** Incidence of Cardiovascular Events in the Two Arms of the Study

	Diabetic (Total=95)		Non-diabetic (Total=115)		*P*-value
	Frequency	Percent	Frequency	Percent	
Positive cardiac scan or exercise test	5	5.26	8	6.96	0.61
Restenosis in angiography	4	4.21	3	2.61	0.52
Acute or subacute MI	1	1.05	2	1.74	0.68
Late MI	2	2.10	1	0.87	0.45
CABG	3	3.16	2	1.74	0.50
Repeat PCI	1	1.05	0	0	0.27
Cardiac death	1	1.05	1	0.87	0.89
Event-Free Survival	87	91.6	109	94.3	0.35

Abbreviations:MI, myocardial infarction; CABG, coronary artery bypass grafting; PCI, percutaneous coronary interventions

Table 6 shows that target vessel failure (TVF) chance increases in diabetic and non-diabetic groups with stent length less than 25 mm, but regarding stent length our result did not reveal any significant difference between the two groups.

**Table 6:** Indicator Variable of Stents and Relation with TVF in Diabetic and Nondiabetic Patients

Stent's indicator	TVF in diabetic group	TVF in nondiabetic group	*P* value
Stent Length (mm)	21.3 ± 3.4	19.6 ± 2.3	0.3
Stent diameter (mm)	2.8 ±0.4	3 ± 0.3	0.52

Abbreviations: TVF, Target Vessel Failure: composite of cardiac death.

As demonstrated in Table 7 mortality rate in our study was 1% which was consistent with TAXUS II (0.8%), Simple II (1%), TAXUS IV (1.4%) and Sirius (1.4%). Mortality rate after using infinium-eluting stent was 2.1% in Simple I trial and 1% in Simple II and after Sirolimus-eluting stent in E Sirius an Sirius trials were 1.4% and 1.1%.

**Table7:** Occurrence of Cardiac Events after Des in Various Trials, after 6 Months Follow up in Diabetic Patients

	TAXUS II	TAXUS IV	TAXUS VI	Sirius	Simple I	Simple II	Our study
**Mortality**	0.8	1.4	0	1.4	2.1	1	1
**CABG**	0.8	ND	0.9	ND	0.4	1.0	2.5
**MI**	2.41	3.5	8.2	2.8	ND	1	3
**TVF**	ND	ND	16	ND	ND	ND	7

Abbreviations: TVF, Target Vessel Failure

## 4. Discussion

Elezi and et al. compared clinical and angiographic outcome after coronary stent placement between diabetic and non diabetic patients in 1998 and reported that diabetic cases had a less favorable clinical outcome ,one year after successful stent placement compared to nondiabetic patients which had a higher chance of revascularization (15).

Diabetic patients have dismal prognosis and unique response to coronary revascularization. Compared with nondiabetic individuals. Also patients with diabetes carry a greatly increased risk not only for sustaining cardiovascular events but also for poorer outcomes associated with CVD, marked by significantly increased mortality (15).

Elezi et. al showed that the relationship between vessel size and the probability of restnosis is nonlinear and complex lesion situated in small vessels (<3 mm) are prone to high risk of restenosis (15). This was in agreement with our study which showed a high percentage of TVF in patients with stent diameter equal or less than 3mm in both groups, although there was no statistically significant difference between diabetic and non- diabetic groups (Table 6).

In this study requirement for CABG after DES increased compared to the other trials as shown in Table 7. This may be due to high percentage of diabetic patients in our study.

Tanigawa et al, in 2002, showed that diabetics who underwent stent placements had a favorable long term clinical outcome which was similar to non diabetics. Such long term event free survival was not statistically different between both groups. They compared140 diabetics (156 lesion) and 169 non-diabetics (187 lesion), and found that target lesion revascularization was not significantly different between the two groups (16).

Both PCI and CABG techniques demonstrate poorer outcomes in diabetics compared to non diabetic patients. In the landmark BARI trial, 57.8% of diabetics undergoing CABG had 10-year survival compared to 45.5% with angioplasty, which had substantially higher repeat revascularization rates (16). This is in agreement with the results of Hannan EL et al (17). when one revascularization modality was compared to the other Furthermore, CABG consistently demonstrated to be more efficacious than PCI. Thus, current guidelines recommend CABG for the treatment of multivessel disease in diabetics (18,19), as the use of DES decreased the need for revascularization.

## 5. Conclusion

Our results show that PTCA and stenting with DES in patients with DM is a successful procedure and the results are comparable to those of non- diabetics in regard to comparing between one and two vessel disease in 6 months and one year follow up.

## Study limitations

The limitation of this study was its small sample size. Thus further researches are required for better interpretation and analysis of the results.
